# Cerebellar sub-divisions differ in exercise-induced plasticity of noradrenergic axons and in their association with resilience to activity-based anorexia

**DOI:** 10.1007/s00429-016-1220-2

**Published:** 2016-04-07

**Authors:** Hermina Nedelescu, Tara G. Chowdhury, Gauri S. Wable, Gordon Arbuthnott, Chiye Aoki

**Affiliations:** 1 Department of Systems Neurophysiology, Tokyo Medical and Dental University Graduate School, 1-5-45, Yushima, Bunkyo-ku, Tokyo, 113-8519 Japan; 2 Center for Neural Science, New York University, New York, NY 10003 USA; 3 Brain Mechanisms for Behaviour Unit, Okinawa Institute of Science and Technology Graduate University, Okinawa, 904-0495 Japan; 4 Department of Neuroscience, University of Pittsburgh, Pittsburgh, PA 15260 USA

**Keywords:** Noradrenergic axons, Cerebellum, Exercise, Activity-based anorexia, Food restriction, Voluntary wheel running

## Abstract

The vermis or “spinocerebellum” receives input from the spinal cord and motor cortex for controlling balance and locomotion, while the longitudinal hemisphere region or “cerebro-cerebellum” is interconnected with non-motor cortical regions, including the prefrontal cortex that underlies decision-making. Noradrenaline release in the cerebellum is known to be important for motor plasticity but less is known about plasticity of the cerebellar noradrenergic (NA) system, itself. We characterized plasticity of dopamine β-hydroxylase-immunoreactive NA fibers in the cerebellum of adolescent female rats that are evoked by voluntary wheel running, food restriction (FR) or by both, in combination. When 8 days of wheel access was combined with FR during the last 4 days, some responded with excessive exercise, choosing to run even during the hours of food access: this exacerbated weight loss beyond that due to FR alone. In the vermis, exercise, with or without FR, shortened the inter-varicosity intervals and increased varicosity density along NA fibers, while excessive exercise, due to FR, also shortened NA fibers. In contrast, the hemisphere required the FR-evoked excessive exercise to evoke shortened inter-varicosity intervals along NA fibers and this change was exhibited more strongly by rats that suppressed the FR-evoked excessive exercise, a behavior that minimized weight loss. Presuming that shortened inter-varicosity intervals translate to enhanced NA release and synthesis of norepinephrine, this enhancement in the cerebellar hemisphere may contribute towards protection of individuals from the life-threatening activity-based anorexia via relays with higher-order cortical areas that mediate the animal’s decision to suppress the innate FR-evoked hyperactivity.

**Electronic supplementary material:**

The online version of this article (doi:10.1007/s00429-016-1220-2) contains supplementary material, which is available to authorized users.

## Introduction

The cerebellum has long been associated with motor control (Holmes 1917) but has also become recognized recently for its participation in cognitive, language and spatial processing (Strick et al. 2009; D’Mello and Stoodley 2015). This structure is extensively innervated by noradrenergic (NA) axons arising from the locus coeruleus (LC), which consists of neurons that become most active when an individual is in a state of vigilance or arousal (Berridge and Waterhouse 2003; Loughlin et al. 1986; Olson and Fuxe 1971; Sara and Bouret 2012; Pickel et al. 1974; Bloom et al. 1971; Mugnaini and Dahl 1975; Hokfelt and Fuxe 1969; Chandler et al. 2013; Aston-Jones et al. 1991). The significance of cerebellar norepinephrine neurotransmitter in motoric control is highlighted by the finding that depletion of cerebellar norepinephrine impairs acquisition of novel locomotive tasks, such as to run on a rod runway (Watson and McElligott 1984), while the decline of the cerebellar norepinephrine system with aging has been shown to retard the rate of acquisition of motor skills (Bickford 1993; Bickford et al. 1992). This age-related decline is ascribed to changes in the density of β2-adrenergic receptors within the cerebellum. Associative motor learning involving the cerebellum, such as delay eyeblink conditioning, also depends on the cerebellar NA system, since norepinephrine is released in the cerebellum precisely during this task and blockade of β-adrenergic receptors in the cerebellum impairs consolidation of this task (Paredes et al. 2009).

Previous work demonstrated that exercise causes an increase in Purkinje cell dendritic field area and total branch length (Pysh and Weiss 1979). Similar to postsynaptic elements including dendritic spines, presynaptic axons of the cerebellar parallel fibers show dynamic structural changes (Carrillo et al. 2013). However, whether exercise evokes structural changes to NA fibers in the cerebellum is a question never before posed but seemed possible, since exercise has already been shown to augment the level of norepinephrine in the locus coeruleus (Dishman 1997) and to evoke changes in norepinephrine metabolism at other terminal fields of LC—the hippocampus and amygdala (Dishman et al. 2000). These exercise-evoked changes have been suggested to confer protection from stress-induced depletion of norepinephrine in the terminal fields (Dishman et al. 2000). There is immense literature indicating that exercise improves learning, memory and cognitive flexibility (for example, (Barrientos et al. 2011; van Praag et al. 2005; Vaynman et al. 2004; Brockett et al. 2015)) as well as resilience to stress (Schoenfeld et al. 2013) through changes in neurotransmitter systems other than the NA system or brain regions other than the cerebellum. We sought to provide complementary data by determining whether mild versus excessive voluntary wheel running activity (WRA) evoked different types of structural plasticity to the NA fibers in the cerebellum.

Neurons of the LC innervate the granule cell layer, the Purkinje cell layer and the molecular layer (Mugnaini and Dahl 1975). We chose to focus our analysis on the molecular layer of the cerebellar cortex, which houses large Purkinje cell (PC) dendritic arbors, because this layer contains the highest level of β-adrenergic receptor-like immunoreactivity within the cerebellar cortex (Aoki et al. 1987). To analyze NA fibers’ structural plasticity following vigorous exercise, we used a paradigm, called activity-based anorexia (ABA). ABA has been used widely for identifying the neurobiological consequences of voluntary excessive exercise (EEX, averaging 15 km/day for rats) that is evoked by temporarily housing animals in an environment with ad libitum access to a running wheel but limited access to food (1 h/day for rats) (Aoki et al. 2012). Fifty years ago, it was shown that when food restriction is combined with wheel access, the majority of the mildly running rats convert to becoming excessive runners, choosing to run, even during the periods of food availability, thereby “self-starving” to death, unless removed from this ABA-inducing environment (Gutierrez 2013; Wable et al. 2015; Routtenberg and Kuznesof 1967). There are a number of theories regarding the reason that food-restricted animals paradoxically become excessive wheel runners, with one being that an innate foraging-like behavior is triggered when starved (Guisinger 2003; Gutierrez 2013). Because hyperactivity, self-starvation and severe weight loss of ABA animals capture the core symptoms of anorexia nervosa, ABA has been used to understand the biological changes evoked within the brain and the body of individuals with anorexia nervosa (Beumont et al. 1994; Casper et al. 2008; Davis et al. 1997). In order to induce mild exercise (EX, ~5 km/day), a separate group of animals had unlimited access to both food and a running wheel. To differentiate the effect of excessive exercise from that of food restriction, cerebellar samples from the EEX and EX groups of rats were compared to samples from age-matched groups of sedentary but food-restricted (FR) rats, and from a control (CON) group of rats that were housed concurrently with ad libitum access to food and no access to a running wheel. Anorexia nervosa is nine times more prevalent in females than in males and emerges during adolescence (ages 14-19) (Kaye 2009). Therefore, we chose to examine the neuroanatomical changes evoked by ABA induction within brains of rats that matched this profile—i.e., adolescent females.

We conducted NA fiber analysis across two sub-divisions of the cerebellum—the D zone of the hemisphere and the A zone vermal modules, because they are functionally distinct (Voogd 2014; Apps and Hawkes 2009; Cerminara and Apps 2011; Apps and Garwicz 2005). For example, the A zone vermal module is composed of alternating zebrin-positive and -negative subzones, each of which receive afferent information from particular regions of the medial accessory olive (Sugihara and Shinoda 2004; Voogd 2014). In contrast, the D zone of the hemisphere has a less organized zebrin pattern, with borders between the subzones that are not easily determined. Moreover, climbing fiber afferents and efferent projections of the hemisphere are distinct from those of the A zone vermal module (Voogd 2014). Their functional subdivisions are dictated by precise inputs onto cerebellar Purkinje cells which, in turn, are the sole output cells of the cerebellum. Thus, the Purkinje cells serve as the nodes for integrating the cerebellum with motor as well as non-motor circuits at all levels (Strick et al. 2009; Sillitoe et al. 2009; D’Mello and Stoodley 2015). Specifically, the longitudinal zones of the cerebellar hemisphere receive little to no input from the spinal cord and are, instead, heavily interconnected to non-motor areas, including the prefrontal cortex that mediates behavioral flexibility (Strick et al. 2009; Stoodley 2015; Balsters et al. 2010). In contrast, the vermal region modules are densely connected with the motor cortex and receive heavy direct innervations from the spinal cord (Coffman et al. 2011; Provini et al. 1968; Strata et al. 2012; Sengul et al. 2014; Reeber et al. 2013). While much is known about the neuronal wiring, less is known about the modules’ contrasting roles in behavior. To date, comparisons of the NA innervation pattern between the vermis and the hemisphere has not been made. Therefore, we also determined whether the NA axonal pattern is different between the two regions in the basal state and whether exercise and/or food restriction evoke changes in the NA innervation pattern differently across the two regions.

We integrated confocal microscopy and Neurolucida digital tracing software to facilitate the acquisition and extraction of structural information about varicosity and NA axons immuno-labeled for dopamine β-hydroxylase (DβH), a marker of norepinephrine-synthesizing neurons and their processes. This approach enabled three-dimensional (3D) reconstruction of spatially distributed NA axonal varicosities from image stacks. We determined voluntary activity-evoked plasticity of NA varicosities, reflecting altered local and global transmission in the molecular layer of the vermis and the hemisphere of cerebellar cortex. Using Voronoi tessellation, we investigated the spatial distribution of the population of varicosities in both regions of interest. Correlation analyses were conducted to identify associations between NA structural plasticity and WRA of individual rats.

Our results suggest distinct modes of operation for LC–NA axons projecting to the cerebellar vermis and hemisphere regions in response to WRA of different intensities, with implications for their differential roles in reducing vulnerability of individuals to ABA.

## Materials and methods

### Subjects

All live-animal procedures were in accordance with the Institutional Animal Care and Use Committees of New York University (A3317-01). Sprague-Dawley female rats of age postnatal day (P) 28 were purchased from Taconic Farms and delivered to New York University.

### Exercise, food restriction schedules, and data collection

**Fig. 1:**
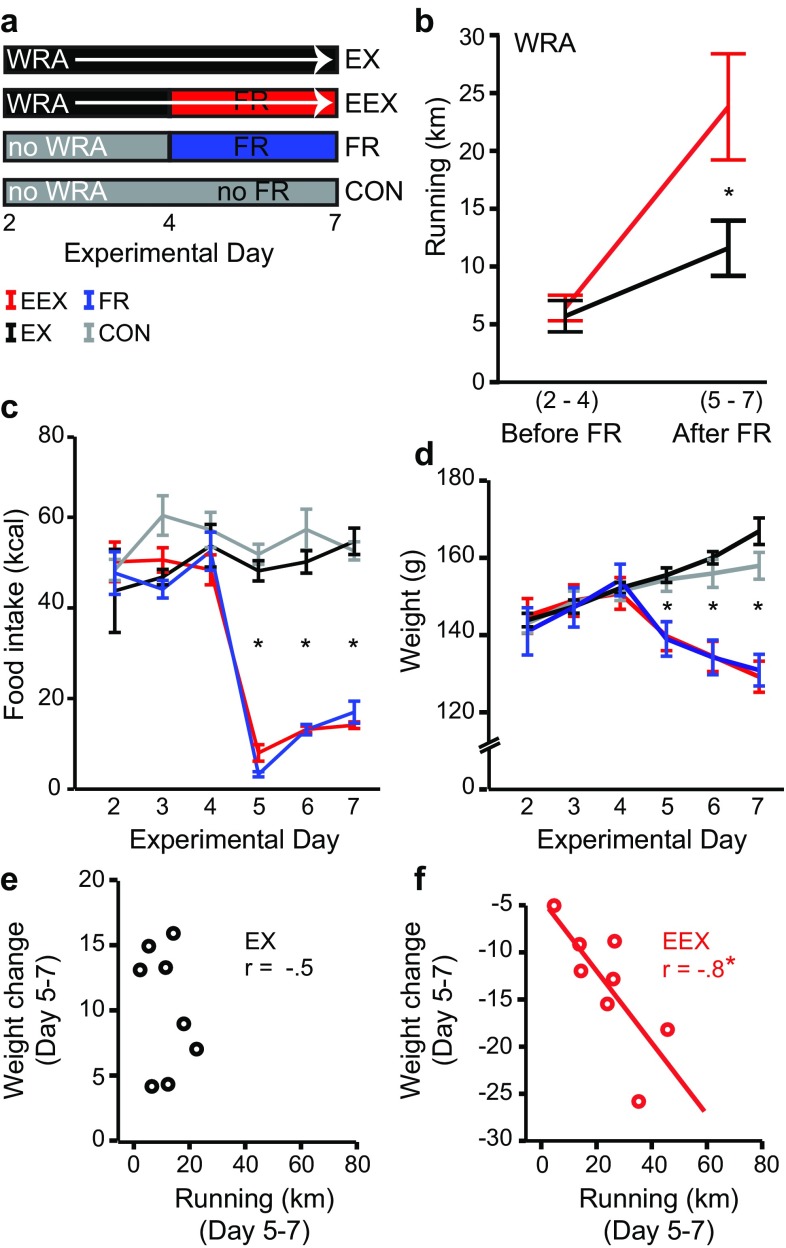
Training paradigm and wheel running activity. **a** Scheme summarizing the training paradigm: mild EX runners (running wheel and ad libitum food access); excessive EEX runners (running wheel with food restriction); FR (no running wheel with food restriction; CON (no running wheel and ad libitum food access). At P44, animals were euthanized prior to the end of the light cycle, thus, before the start of the 8th day. **b** Total running activity before and after food restriction, starting with Day 2. Note the increase in running activity for both EX and EEX animals after Day 4, with significantly more increase in running behavior for the EEX group (*asterisks* indicate *P* < 0.01, *N* = 8 per group). **c** Total food intake in relation to each experimental day (*asterisks*, significant denote one-way ANOVA *P* < 0.001, post hoc LSD *P* < 0.05 for EX/CON vs EEX/FR, *N* = 8 rats per group). **d** Weight changes with respect to each experimental day, showing significant changes in EEX rats after Day 4 (*asterisks*, significant one-way ANOVA *P* < 0.001, post hoc LSD *P* < 0.05 for EX/CON versus EEX/FR, *N* = 8 per group). *FR* Food restriction, consisting of 1 h of food access only during the first hour of the dark cycle. *Error bars* ± 1 SEM. **e**, **f** Weight change and running activity after food restriction. The *solid red* trend *line* and *asterisk* in *panel* **f** indicates significance of Pearson correlation (*P* < 0.05)

Upon arrival at P28, rats were individually housed with ad libitum water and rat chow on a 12 h light / dark cycle. Subsequently, four groups of eight rats participated in the following training paradigms; the mild exercise group (EX, ad libitum food and running wheel access), excessive exercise group (EEX, 1 h/day food access and ad libitum running wheel access), food restriction-only group (FR, food access for 1 h/day and no wheel access) and control group (CON, ad libitum food access, and no running wheel access) (Fig. 1a). After the acclimation period from P28 to P35, individually housed EX and EEX animals were moved to a new home cage connected to a running wheel (Med Associates, Inc., St. Albans, VT, USA). Voluntary wheel running activity (WRA) was measured starting P36 up to P44. For EEX and FR animals, food restriction began at P40, where animals received unlimited access to food for only 1 h/day at the onset of the dark cycle (Fig. 1a). Each day, the body weight, food intake and WRA were measured prior to the onset of the dark cycle. WRA data were also collected automatically and continuously using the software of Med Associates (Chowdhury et al. 2015).

### Tissue preparation

At P44, corresponding to the 8th day of wheel access of the EX and EEX group and the 4th day of food restriction for the EEX and FR group, animals were anesthetized using urethane (34 %; 0.65–0.85 ml/185 g body weight) via intraperitoneal injection, then euthanized by transcardial perfusion with a fixative consisting of 4 % paraformaldehyde in 0.1 M phosphate buffer (pH 7.4) over 10 min at a flow rate of 50 ml/min. After decapitation, brains were quickly removed from the skull. Brains were prepared into 2 mm-thick coronal slabs with a razor blade. The following day, coronal sections including the cerebellum were cut with at a thickness set at 50 μm, using a Leica VT1000 M vibratome (Leica Microsystems GmbH, Wetzlar, Germany). DβH, the enzyme that converts dopamine to norepinephrine, served as a tool to immunolabel NA nerve terminals in the cerebellum (Hartman et al. 1972; Swanson 1976; Hokfelt et al. 1984). To visualize the NA axonal plexus in the cerebellum, a cocktail consisting of a mixture of two mouse monoclonal antibodies directed against DβH were used (Chemicon catalog number C4M020, MAB308, lot 23020627, at a dilution of 1:1000 and Fitzgerald catalog number 10-D60, [MC antibody M26087], at a dilution of 1:50). This mixture of two monoclonal antibodies was used, so as to maximize detection of antigenic sites within aldehyde-fixed tissue. This cocktail was suspended in 0.01 MPB containing 0.9 % sodium chloride (0.01 M PBS, pH 7.4) together with 0.3 % Triton X-100 to permeabilize the tissue, 0.05 % sodium azide to retard bacterial growth and 1 % bovine serum albumin to reduce non-specific immunolabeling. Free-floating coronal sections containing both the locus coeruleus (LC) and cerebellar cortex were incubated overnight at room temperature, on a shaker in the buffer containing primary antibodies. On the following day, the excess unbound primary antibodies were removed by thoroughly rinsing the tissue with cold 0.01 M PBS (pH 7.4). Tissue sections were incubated with biotinylated horse anti-mouse-IgG secondary antibody (Vector Laboratories, Catalog # BA-2000, Burlingame, CA, USA; 1:200) diluted in PBS containing 1 % bovine serum albumin and 0.05 % sodium azide for 4 h on a shaker at room temperature. Next, tissue sections were thoroughly rinsed in PBS and incubated in PBS containing biotinylated HRP-avidin complex (ABC Elite kit, Catalog # 6100, Vector Laboratories). The immunolabel was visualized by the HRP-DAB procedure, with 3,3′-diaminobenzidine HCl (DAB) and H_2_O_2_ as substrates. DAB solution was prepared by dissolving 1 DAB tablet (10 mg, Sigma #D-5905) in 43 ml PBS and adding 4.3 μl of 30 % H_2_O_2_. Tissue sections were incubated in the DAB solution for 10 min. The HRP reaction was terminated by rinsing tissue sections in PBS. Sections were mounted and dried before cover-slipping with Permount mounting medium.

**Fig. 2:**
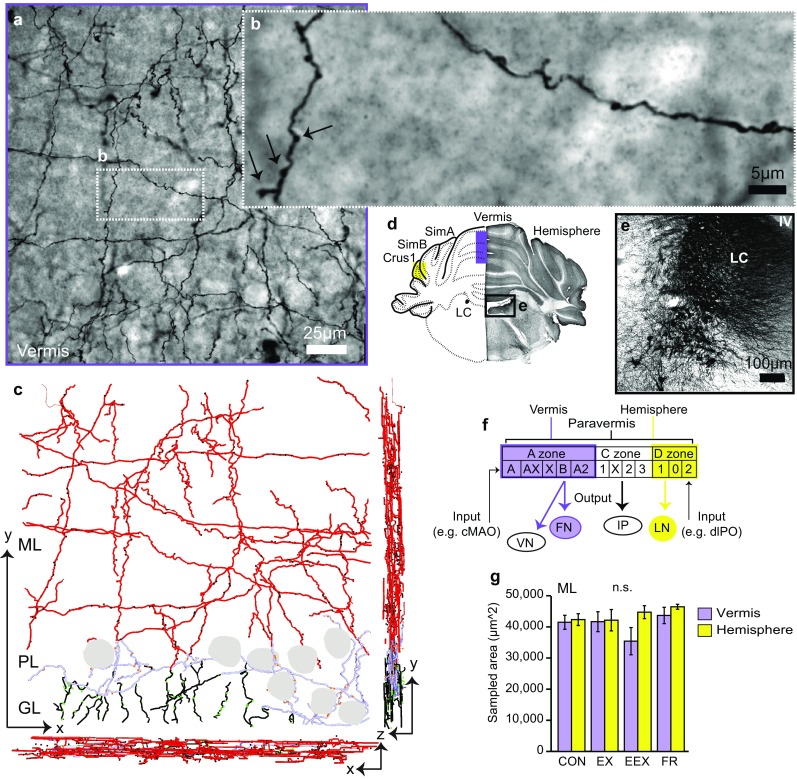
Confocal style stack and systematic 3D reconstruction of NA fibers. **a** Coronal view of the vermis NA axonal plexus in the ML, PL, and GL, revealed by immunohistochemical labeling, using anti-DβH antibodies, and visualized with DAB. **b** Enlarged *inset* from **a** demonstrates swollen varicosities (*black arrows*) along NA fibers. **c** Neurolucida-reconstructed NA axons from the image stack in (*A*) viewed in the *x*, *y* plane, and orthogonal *x*, *z* and *y*, *z* planes after correcting for shrinkage in the *z* plane. Small filled circles are markers representing the NA varicosity location in three-dimensional space. Note that the marker size does not reflect actual biological varicosity sizes. *Gray* contours depict locations of Purkinje cell bodies. **d** Low magnification (10×) tiled image stained for DβH and schematic of the coronal slice showing the sampled vermis (*purple fill*) and hemisphere (*green fill*) regions. **e** Darkly stained LC NA neurons demonstrating the specificity of the DβH antibody. **f** The rationale for investigating the vermis and hemisphere regions depicting distinct output and input connectivity to the vermis (*A* *zone*) and hemisphere (*D zone*), based on previous works (Apps and Hawkes 2009; Sillitoe et al. 2009; Sugihara and Shinoda 2004; Voogd and Ruigrok 2004). The *boxes* below each zone are further subdivisions with specific input and output connectivity (example shown by input/output arrows). **g** Summary plot showing consistently sampled areas across groups, *N* = 8 rats per group except *N* = 7 for the hemisphere of EEX and FR groups. Error bars are SEM, n.s. (not significant; one-way ANOVA *P* > 0.05). *DβH* dopamine β-hydroxylase, *DAB* 3-3′ diaminobenzidine, *cMAO* subnucleus b, *dlPO* dorsal lamella of the principal olive, *ML* molecular layer, *PL* Purkinje cell layer, *GL* granule cell layer, *LC* locus coeruleus

Labeling was intense for the cell bodies in the LC (Fig. 2e), and absent within nuclei containing the dopaminergic cell groups of substantia nigra, ventral tegmental area, hypothalamus and also absent in the nuclei containing the serotonergic cell groups of the median raphe and dorsal raphe. Fibers and varicosities were abundant in the cerebellar cortex (Fig. 2a–d).

### Sampling and confocal microscopy

Slides containing labeled NA axons in cerebellar tissue samples were imaged with an LSM-710 confocal microscope using the Photon Multiplier Tube (PMT). Our tissue samples were not fluorescent. However, the confocal laser (488 nm) was used to scan the tissue using the PMT to digitize the image and create z-stacks (Fig. 2a, b). With this procedure, confocal-style stacks of darkly non-fluorescently labeled varicosities along axons were acquired from the molecular layer of coronal tissue sections corresponding to the following coordinates: −9.96 to −10.44 mm Bregma, −0.96 to −1.44 mm Interaural (Paxinos and Watson 2005). Vermis coronal sections were imaged between 0.1–1 mm from the midline and 0.5–2.5 mm from the dorsal surface, while for the hemisphere, the images were obtained 4.5–5.4 mm from the midline and 0.1–2.4 mm from the dorsal surface (Paxinos and Watson 2005). Sampling in the anterior vermis region was equivalent to longitudinal zone A, whereas sampling in the hemisphere was acquired from longitudinal zone D (Fig. 2f). Three-dimensional (3D) volumetric stacks of these regions containing labeled NA fibers were captured with the following image acquisition parameters; 63× oil objective lens with a numerical aperture of 1.4, optical sectioning with a z-step size of 0.25 μm, x/y image size of 224 × 224 μm (for CON, EX and EEX) and 224 × 224.9 μm (for FR tissue), and a resolution of 2048 × 2048 pixels. The microscope was set to acquire stacks of the entire thickness of the tissue. However, for consistency across samples, a set thickness of 10 μm was used during the digital extraction phase and reconstruction of NA axons. This was achieved by detecting the middle of the stack and using only the portion 5 μm above and 5 μm below this middle point for NA axonal reconstruction. The remaining optical slices were eliminated and excluded from the analysis. This trimming step was necessary as the tissue blocks were vibratome sections with the thickness setting of 50 μm, which became thinner during the dehydration process following DAB visualization. This trimming step ensured standardization of the reconstructed volumes of the NA plexus across samples. In addition, a shrinkage factor in the z-axis was determined by measuring multiple processed sections on the glass slide with the confocal microscope. Then, the shrunken tissue sections were compared to the actual thickness cut by the vibratome, yielding a shrinkage factor of 1.5.

The sampled zone was always within the molecular layer, only, of the two cerebellar regions and across the four experimental groups, EEX, EX, CON, and FR. This resulted in slight differences (<15 %) in the sampled volumes per group, due to the different extent of the Purkinje cell layer and granule cell layer that ‘invaded’ the scanned volume. The Purkinje cell layer and the granule cell layer were excluded from the volume to be used for NA axon structure analysis (Fig. 2c, g).

### Reconstructions

The trimmed digital stacks containing information about the DβH-immunoreactive NA varicosities and axonal plexus were individually loaded into the Neurolucida (MBF Bioscience, VT, USA) tracing software. To eliminate bias during data collection, the identity of the experimental group to which the digital stacks belonged was kept blind until the statistical analysis step. NA fibers were manually traced from stacks using a digital pen and a Wacom Cintiq video tablet. To denote NA axonal varicosities, each time a swelling was encountered, a marker was placed along the axonal fiber denoting the precise location of the varicosity in 3D space (Fig. 2c). The reconstructed axons were corrected for tissue shrinkage in the z-axis, the plane in which the majority of shrinkage occurs.

### Quantitative analysis of anatomical data

Each animal’s cerebellar tissue had a reconstructed NA axonal profile with the precise number and location of each varicosity, recorded while kept blind to the animal’s rearing condition. In addition, inter-varicosity intervals and the nearest neighboring varicosity distances (NNVD), as well as the total number of varicosities, axonal fiber numbers and lengths were the obtained from Neurolucida Explorer (MBF Bioscience, VT, USA). Fragment varicosity density was computed by dividing the number of varicosities per axon fragment over the length of each fragment, while total varicosity density was obtained by calculating the number of varicosities per sampled volume. Additional quantitative analyses and graphical representations of distribution curves and Voronoi *tessellation* were obtained using functions from Matlab. We present the outcome of each of these anatomical analyses separately under Results and also summarize the results in Table 1 and pictorially in Fig. 9.

**Table 1:**
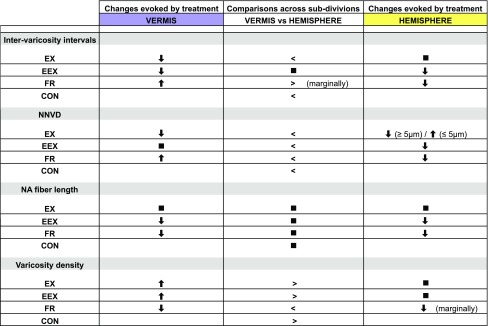
Summary table summarizing findings

Arrows represent changes evoked by treatment relative to control rats. Greater than or less than signs denote NA structural changes in the vermis compared to the hemisphere. Solid filled squares reflect no difference in NA plasticity relative to control animals

### Statistical analyses

Behavioral analyses were conducted, using eight animals per condition. Statistical analyses of the NA varicosities and axons in the vermis were conducted upon 7 or 8 rats per condition (8 CON, 8 EX, 7 FR and 8 EEX). Statistical analyses for the hemisphere were conducted using tissue from 8 CON rats, 8 EX, 7 FR and, 7 EEX. One EEX tissue of the hemisphere could not be analyzed, due to damage to the glass slide. Simple, Student’s *t* test was used to compare the mean running activity for the EX and EEX groups, as well as the differences in NA axonal structures between the vermis and the hemisphere regions, using *N* = animal number. Mean body weight and food intake were compared with a one-way ANOVA and followed with post hoc LSD). Histograms were examined to assess normal distributions and the Levene’s test was performed to confirm equality of variances between groups. A 2-by-2 factorial ANOVA was used to compared the mean running activity between EX and EEX groups before and after food restriction. Pearson’s correlations were computed between total running activity or weight change and NA inter-varicosity interval, fragment lengths, and varicosity density using *N* = animal subjects. To compare the cumulative frequency distributions of NA structural measurements, non-parametric analyses with the Kolmogorov–Smirnov (K–S) test were performed, using *N* = NA structure. Non-parametric Kruskal–Wallis test and Bonferroni corrections were performed to achieve comparisons of individual group’s mean values of NA varicosity density, using *N* = NA structure. Our rationale for using *N* = NA structure for these analyses is based on the observation that the vesicular release of neurotransmitters from varicosities is independent of neighboring varicosities (Ryan et al. 1993; Harata et al. 2001).

## Results

### Wheel access evokes voluntary running while food restriction evokes excessive voluntary running

It was previously shown that the combination of food restriction plus wheel access induces excessive wheel running which, in turn, exacerbates weight loss beyond that expected from food restriction alone (Routtenberg and Kuznesof 1967; Gutierrez 2013; Aoki et al. 2012). This response pattern was also observed for the cohort of rats in our current study.

We created four groups for this study: excessively running rats (EEX), which were given wheel access and food-restricted; mild runners (EX), which were not restricted of food access; control group (CON) that had no access to a wheel and were not food restricted; and a group that was food restricted (FR) without wheel access (Fig. 1a). We tracked the animals’ daily wheel running activity (WRA), food intake and weight change before and after food restriction, which began on the fourth experimental day for the FR and EEX group, at postnatal day 40, until P44 (Fig. 1a–d).

Our results confirm that food restriction causes rats to run significantly more than by wheel access alone (Fig. 1b), verified by a factorial ANOVA (*F* (1,28) = 18.2, *P* < 0.001 and significant interaction (*F* (1,28) = 4.44, *P* < 0.05). The increased WRA coincided with the experimental day after food access began to be restricted (Fig. 1c) and was accompanied by weight loss of the EEX rats but not of the EX rats (Fig. 1d). Pearson’s correlation analysis demonstrated that the extent of weight loss of the EEX rats correlated strongly with the degree of hyperactivity, indicating that suppression of WRA minimizes excessive weight loss (Fig. 1f). In contrast, the weight change of the EX group did not correlate with their WRA (Fig. 1e).

EX animals from Day 2 to 7 averaged a WRA of approximately 3.5 km/day. The day-by-day differences in running activity between the EX and EEX animals were statistically significant at Day 5, 6 and 7, (Student’s *t* test, *P* < 0.01). The largest WRA difference occurred on the last day, where WRA averaged 4.6 km for EX rats and 19 km for EEX rats (Online Resource 1). The difference in WRA exhibited by the two groups of animals provided an opportunity to examine the effects of voluntary WRA levels on the structural plasticity of the NA system.

### Mapping of NA axons following voluntary running

NA varicosities are sites of vesicle and DβH accumulation, and are thus reasonable to consider as sites specialized for norepinephrine synthesis and release (Aoki 1992; Descarries and Mechawar 2000; Papadopoulos et al. 1987a; Harata et al. 2001; Ryan et al. 1993). Specificity of the antibody for detecting the NA system within the tissue prepared from this cohort of animals was confirmed by the intense, selective labeling of NA cell bodies in the LC (also referred to as group A6, Fig. 2d, e) and notable NA fibers in the cerebellum (Fig. 2a–b). The only other NA cell groups with intense labeling were nuclei of the medulla, i.e., the A1 and A2 cell groups (Dahlstrom and Fuxe 1964) and the C1 adrenergic group, (Milner et al. 1989; Hokfelt et al. 1974). By combining scanning confocal microscopy with digital image reconstruction, we acquired virtual tissue volumes, capturing 3D structural information of NA axons and of their varicosities (Fig. 2c and video Online Resource 2). These 3D reconstructions provided a means for quantitative analysis and demonstrated varicosity along NA axons wrapping closely around contours of PC somata, densely coursing through the granule cell layer and extending into the molecular layer (Fig. 2c). Since it is established that the anterior vermis and lateral ansiform lobule of the hemisphere have different input–output connectivity (Cerminara and Apps 2011; Coffman et al. 2011; Voogd 2014; Provini et al. 1968; Voogd and Glickstein 1998) (summarized in the “Introduction” section), we analyzed NA varicosities and axons in longitudinal zones A (anterior vermis) and D (hemisphere) separately (Fig. 2d, f). The sampled zones were consistently within the molecular layer, only, of the two cerebellar regions and across the four experimental groups; EEX, EX, CON, and FR. (Fig. 2c).

### Inter-varicosity intervals are altered differently by exercise versus food restriction and across the vermis versus hemisphere

#### The vermis

**Fig. 3:**
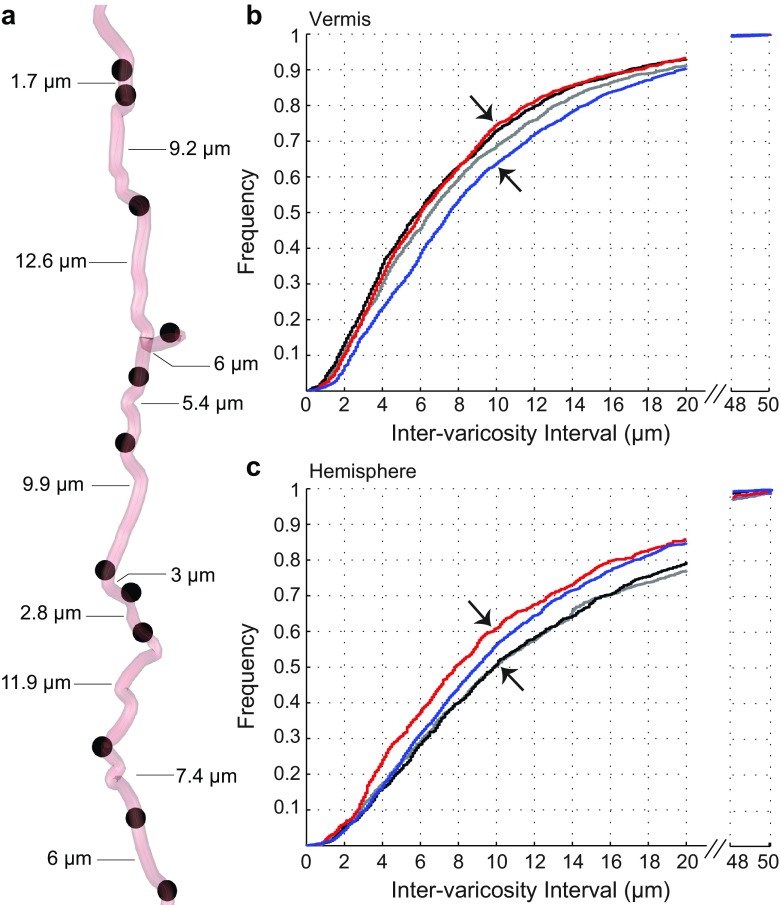
Inter-varicosity intervals along NA fibers following running activity. **a** An example of a NA axon fiber with 12 varicosities, separated by different axonal lengths, measured with Neurolucida Explorer. **b**, **c** Graphical representation of the normalized cumulative frequency distributions of inter-varicosity intervals for the vermis and the hemisphere’s NA axons of EX (*black*), EEX (*red*), FR (*blue*) rats, relative to controls (CON, *gray*)

In the vermis, the cumulative frequency distributions of inter-varicosity intervals for NA axons of EX and EEX groups were shifted to the left, relative to the corresponding distribution of CON (Fig. 3b). These differences across the groups were readily apparent for inter-varicosity intervals of 10 μm or smaller (arrows, Fig. 3b). A pairwise comparison of the distributions using the K–S test confirmed that the higher frequency of shortened inter-varicosity intervals of the EEX and EX tissue, relative to CON, were statistically significant (*P* < 0.05). For CON rats, the median of the inter-varicosity distance distribution was 6.6 μm, while the median values were 5.9 μm for EX and 6.0 μm for EEX rats. These leftward shifts of the distribution curves indicate a higher frequency of reduced intervals between varicosities, and that norepinephrine-containing varicosities along axons are closer to each other in the vermis of EX and EEX rats, compared to CONs’ (Fig. 9). The reduction in inter-varicosity interval indicates that NA axons of EX and EEX animals are capable of releasing more NE specifically along the axon’s path than CON animals. A pairwise comparison K–S test indicated that the distributions of varicosities along axons of the EX and EEX rats were not different (*P* > 0.05), implying that the restructuring of NA fibers in the vermis may not be affected by the amount of exercise.

A pairwise K–S test (*P* < 0.001) confirmed that the distribution of FR rats was different from that of CONs, EEXs and EXs in the vermis. The vermis of FR rats exhibited an overall increase in the inter-varicosity interval (FR group, median 7.2 μm) compared to the CON, EX and EEX groups, resulting in a right shift of the distribution curve for FR rats (Figs. 3c, 9; Table 1). The frequency of short intervals is lower for the FR, compared to CONs, EXs and EEXs and this difference across the groups is most readily apparent for the range of inter-varicosity intervals that are 10 μm or smaller (Fig. 3b). This suggests that exercise prevented the effects of food restriction on the NA structure in the vermis.

#### The hemisphere

In contrast to the vermis, where exercise and food restriction had opposite effects, the cumulative frequency distribution of inter-varicosity interval in the hemisphere was shifted to the left for tissue of FR, but not altered for the EX tissue. Moreover, the leftward shift of EEX’s cumulative frequency distribution, in comparison to the CON, was more robust in the hemisphere (Fig. 3c) than in the vermis (Fig. 3b). These shifts indicate that varicosities along axons were closer to each other for the EEX and FR groups, with the effect being greater for EEX (pairwise K–S test: EEX vs CON, *P* < 0.001, medians 7.8 vs 10.0 μm; and FR vs CON, *P* < 0.001, medians 8.6 vs 10.0 μm). The distribution curves of EX (median, 10.0 μm) and CON (median, 10.0 μm) rats were confirmed to be the same by the K–S test (*P* > 0.05). The differences across the groups were most prominent at inter-varicosity distance of 10 μm (arrows, Fig. 3c). What the FR and EEX groups have in common and is not shared by the EX group is food restriction. In summary, the reorganization of the NA system in the hemisphere was affected by food restriction and not by exercise (Fig. 9; Table 1). The greater effect exhibited by EEX rats than by FR rats suggests that excessive exercise may have generated greater stress associated with greater negative energy balance.

### Spatial arrangement of varicosities, independent of axons, is altered by environmental conditions and anatomical region

**Fig. 4:**
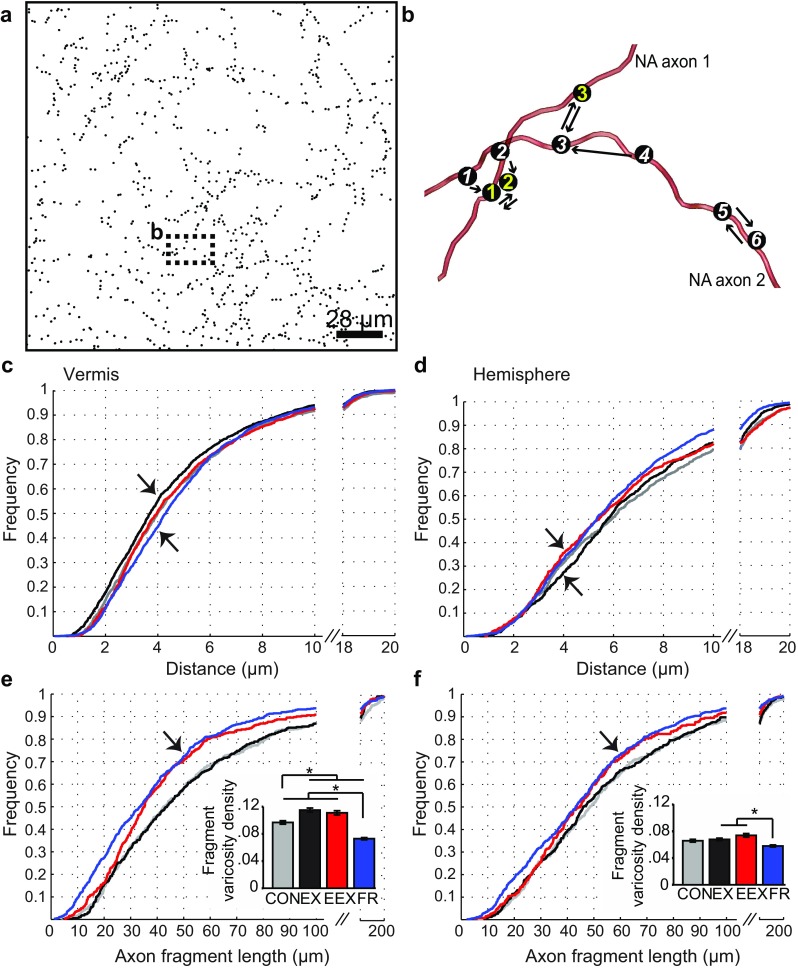
The nearest neighboring varicosity distance (NNVD) of NA varicosities of the three experimental groups. **a** Demonstration of the NA varicosities’ spatial organization, independent of their axon fibers, emphasizing the global release of NE. **b** Scheme showing the method of determining the NNVD among the NA varicosity population. Each *arrow* indicates the nearest varicosity from each originating varicosity. Note that the nearest neighboring varicosities are not always reciprocal. For example, even though varicosity 3 on axon 2 is closest to varicosity 4 on axon 2 (*single arrow*), the nearest neighbor of varicosity 3 on axon 2 is not varicosity 4 on axon 2 but is varicosity 3 on axon 1. The double arrows between varicosity 3 on axon 1 and varicosity 3 on axon 2 are examples whereby the two varicosities are reciprocally closest to each other. **c**, **d** *Graphical* representation of the cumulative frequency distributions of NNVDs for the vermis and the hemisphere NA axons of EX (*black*), EEX (*red*), FR (*blue*) rats relative to controls (CON, *gray*). *Insets* show summary plots of the distribution values. **e**, **f** Averaged cumulative frequency curve for NA fragment lengths in the vermis and the hemisphere

To understand the population-level effect of norepinephrine’s putative release sites, independent of their position along axon fibers, we measured the nearest neighbor varicosity distances (NNVD) (Fig. 4a, b). NNVD values are approximately half of those for the inter-varicosity intervals for all conditions, suggesting that the closest varicosities are found by jumping across neighboring axons (Fig. 9).

#### The vermis

In the vermis, cumulative frequency distribution analysis of NNVDs for norepinephrine release sites were qualitatively similar to what was observed for inter-varicosity distances along axons. The cumulative frequency distribution curve was shifted to the left for EX rats and shifted to the right for the FR group, relative to the CON (Fig. 4c). This difference among the groups was pronounced in the range of NNVDs that were 4 μm or shorter (Fig. 4c, arrows). The CON (median, 4.0 μm), FR (median, 4.1 μm) and EX (median, 3.7 μm) distribution curves were statistically significantly different from one another, as revealed by the K–S test (*P* < 0.05), even though the medians were of similar value. This significance in the differences of the distribution pattern arises from differences in the shape of the distribution and variances (CON 13.4, EX 10.3, FR 7.8). In contrast to the mild WRA evoked by the EX group of rats, WRA exhibited by EEX rats did not evoke detectable population-level varicosity rearrangements in the vermis, relative to CON rats (Fig. 4c) (K–S test, *P* > 0.05). This lack of change by the EEX rats was also a pattern observed by measuring inter-varicosity distances along axons.

#### The hemisphere

In contrast to the vermis, which showed no EEX effect, the hemisphere’s NA varicosities exhibited reductions in the NNVDs for the EEX (median, 5.4 μm) and FR (median, 4.9 μm) group, when compared to tissue of CON (median, 6.0 μm) rats, as indicated by the leftward shift of the NNVD distribution curve (Fig. 4d) and the K–S test outcome (*P* < 0.05). This outcome is congruent with the results of the inter-varicosity intervals along axons (Fig. 3c). Specifically, the NNVD curve of the EEX group was shifted to the left for distances ≥4 μm (arrows), relative to CONs (Fig. 4d). Relative to CONs, an even more significant NNVD distribution curve shift was exhibited by the FR group, (*P* < 0.001, K–S test), especially in the range of 4–10 μm. Taken together with the results regarding the inter-varicosity intervals along axons for the hemisphere, these data suggest that food restriction is the causal factor evoking NA varicosities to become closer to one another in the hemisphere (Fig. 9).

In addition to measuring inter-varicosity intervals and NNVDs, we also quantified the total number of varicosities. The mean total number of varicosities across groups was not statistically significantly different (one-way ANOVA, *P* > 0.05). This highlights the importance of having analyzed the finer inter-varicosity and NNVD measurements.

### Alteration of NA fiber lengths is dependent on activity levels

#### The vermis

In the vermis, the higher frequency of shorter inter-varicosity intervals along NA fibers, without a concomitant higher frequency of the low NNVD values of EEX animals (Figs. 3b vs 4c) raised the possibility that excessive exercise might have caused atrophy or shrinkage of NA axons with the concomitant loss of varicosities. Indeed, the fiber length distribution within the vermis region was shifted to the left for EEX rats, relative to the CON and EX groups’ (Fig. 4e), indicating that excessive exercise evoked a higher frequency of shorter NA fiber segments, especially of ranges between 40–70 μm (K–S test, *P* < 0.001). The leftward shift was also observed for the FR group, indicating the emergence of shorter fiber segments (Fig. 9).

If the decrease in inter-varicosity interval reflected shrinkage of NA axons, then the density of varicosities along axon fragments (fragment varicosity density) might be expected to become elevated. In order to test this idea, we examined the fragment varicosity density, averaged across the sampled NA fragments in the vermis. A Kruskal–Wallis with Bonferroni correction (*P* < 0.001, *N* = NA axons, CON: *N* = 381; EX: *N* = 389; EEX: *N* = 382; FR: *N* = 459) confirmed that the axon varicosity density for active rats (EX and EEX) were, on average, higher than those of CONs (inset, Fig. 4e). These results indicate that WRA, whether mild or excessive, elicits a rearrangement of vermis varicosities along axons. For the EEX group, the increase in fragment varicosity density provides further support for the idea that the neighboring varicosity releasing sites may have become closer to one another due to NA axon shrinkage. The absence of change for the EEX group by NNVD could be because loss of varicosities concomitant with atrophy of the NA axons caused varicosities along different fibers to become further apart (Fig. 9). For the EX group, for which analysis of NNVD and inter-varicosity intervals showed congruent changes, together with no change in fragment length distribution, the release sites may have become closer to one another due to the addition of new varicosities, rather than due to NA axon shrinkage (Fig. 9).

The total varicosity density (number of varicosities encountered per unit volume) and total axon lengths (summation of the lengths of all axon fragments) in the sampled vermis volumes were not significantly different across groups (one-way ANOVA, *P* > 0.05). These results may be due to the greater effect of training condition on individual NA axons than on the total average varicosity and axon population. One way in which the frequency of shorter axon fragments may have increased for the FR and EEX, without changes in total axon lengths, is that FR and EEX promoted sprouting of new axon fragments at newly added branch points, while also leading to the shrinkage of axons elsewhere (Fig. 9).

#### The hemisphere

In the hemisphere, mild WRA of the EX group had little effect on the NA fragment lengths or varicosity density along NA fragments (Fig. 4f). In contrast, the EEX group elicited a leftward shift in the distribution of NA axon fragment lengths, possibly due to shrinkage of existing NA fibers (Fig. 9). Food restriction affected both NNVD and NA fragment lengths in the hemisphere (Fig. 4d, f). This effect was confirmed by the K–S test *(P* < 0.001), whereby the FR group demonstrated shortened NNVDs, a higher frequency of shorter NA fibers, relative to the CON’s distribution, and a lower axon fragment varicosity density relative to active rats (inset, Fig. 4f). These results suggest that FR elicits new varicosities to emerge along newly sprouted shorter NA fibers, leading to a higher frequency of low NNVD values. Alternatively or in addition, FR may have elicited shrinkage of existing NA fibers. A one-way ANOVA revealed that the total varicosity density in the sampled hemisphere volumes was significantly different across groups (*P* < 0.01), with the FR group exhibiting a greater varicosity density relative to all other groups (post hoc, Tukey’s HSD *P* < 0.05). In addition, the total axon length was also greater only for the FR group (one-way ANOVA *P* < 0.001, post hoc Tukey’s HSD *P* < 0.01), which is consistent with the idea that shorter fragments have been added. Therefore, emergence of new varicosities along newly sprouted fragments is likely the case. In addition, some varicosities along pre-existing NA axons are likely to have atrophied, leading to the lower fragment varicosity density along longer NA fibers (inset to Figs. 4f, 3c, summarized in Fig. 9).

### Differences in NA axon structure between vermis and hemisphere

Having observed differences in NA axons’ responsiveness to food restriction, mild exercise and excessive exercise across the vermis and hemisphere (summarized in Table 1), we next determined how the two regions differ in structural plasticity. While the average NA axon fragment lengths in the vermis and hemisphere are the same for EX, EEX and FR (*P* > 0.05, Student’s *t* test), the NNVDs were shorter in the vermis for all three conditions (*P* < 0.005, Student’s *t* test). The inter-varicosity intervals along axons were also shorter in the vermis of EX (*P* < 0.005, Student’s *t* test), but not for EEX (*P* = 0.92, Student’s *t* test) and marginally less for FR (*P* = 0.053, Student’s *t* test). The fragment varicosity density was greater in the vermis of EX and EEX rats (*P* < 0.05, Student’s *t* test) and FR rats (*P* < 0.005, Student’s *t* test) (Fig. 4e, f, insets).

**Fig. 5:**
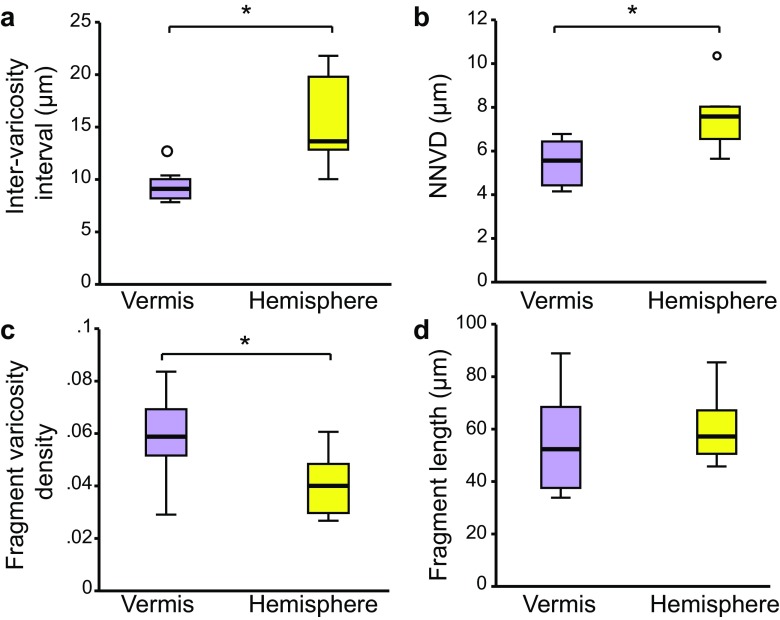
NA innervation of the vermis and the hemisphere at basal state. **a** Inter-varicosity interval, **b** NNVDs, **c** fragment varicosity density and **d** average fragment length compared between vermis and hemisphere in sedentary CON rats. The *boxes* depict the minimum and maximum sample range (*whiskers*), interquartile range (*box*) and median (*horizontal bar*). *Asterisks* depict statistical significance *P* < 0.05, *t* test; *small circles* depict outliers that were included in the statistical tests

Due to these regional differences in NA structure following training, this led us to ask whether the NA system of the vermis and hemisphere were already different prior to training. Student’s *t* test of CON tissue (Fig. 5) revealed that intervals between varicosities along axons are shorter for the vermis (*P* < 0.01, *N* = 8 animals), that varicosity density along axon fragments is higher for the vermis (*P* < 0.05, *N* = 8 animals), and that NNVDs are shorter for the vermis (*P* < 0.01, N = 8 animals). The average axon fragment length was not statistically significant (Fig. 5d, *P* > 0.5, Student’s *t* test). In addition, data from the Allen Institute Online Mouse Connectivity Atlas (http://www.connectivity.brain-map.org) also demonstrates more innervation of NA axons in the anterior vermis region than in the hemisphere (Online Resource 3). Taken together, these data suggest that the cerebellar vermis at basal state has higher NA innervation, compared to the hemisphere region.

### Local NA varicosity clusters are independent of training and cerebellar zone but are present even in the basal state

**Fig. 6:**
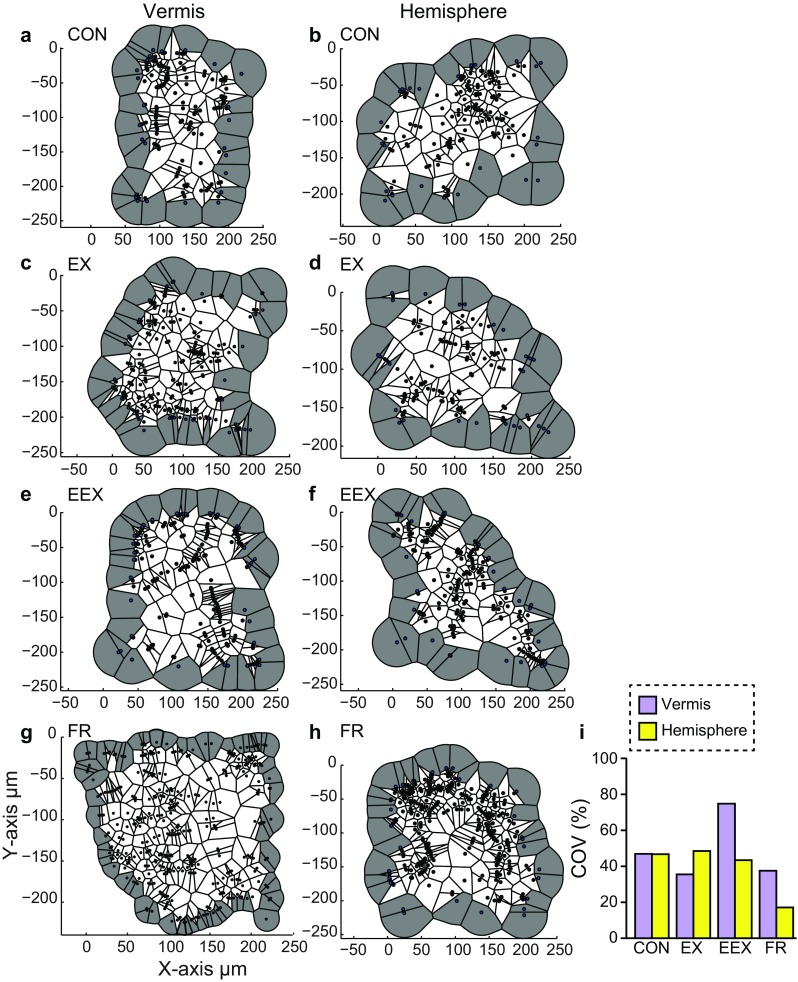
The Voronoi tessellation which measures the spatial distribution of varicosities, demonstrates that NA varicosity clusters are present in the cerebellum of CON rats, and that this clustering is altered following wheel running in an anatomically specific manner. **a**–**f** Examples of Voronoi polygons from regions of interest in the vermis and the hemisphere of CON, EX, EEX and FR rats. *Gray zones* indicate varicosities in the outer-most margins that were excluded from the calculation of averaged values of Voronoi polygons. Voronoi polygons are contiguous, filling the space around each NA varicosity without overlap, hence the term *tessellation*. Voronoi *maps* are shown in the X/Y plane and consist of all varicosities from the confocal stack collapsed onto a 2D plane. **i** Coefficient of variation (COV) reporting the percentage of varying Voronoi polygon areas, which indicates that the NA varicosity population is clustered, as a value of 0 % reflects no clustering in the population

While the NNVD provides information about the next nearest neighboring varicosity and averaged density within the entire surveyed neuropil, it does not provide a measure about localized clusters. To better study the spatial distribution of varicosities, we used the Voronoi (or Dirichlet) tessellation procedure, which focuses on the region of space occupied by the closest varicosity to any other varicosity by discretizing the space in the area of interest (Duyckaerts et al. 1994). We determined whether NA varicosities cluster in response to voluntary exercise, by performing the Voronoi tessellation on the same population of varicosities that underwent NNVD analysis. We examined the effects of voluntary exercise and food restriction on the spatial rearrangement of varicosities by using a map of the areas of the Voronoi polygons as an index to compare across groups. A perfectly homogeneous spatial distribution of varicosities would result in Voronoi polygon areas of the same size. Instead, we detected small and large polygons, even in the CON group, within the vermis and the hemisphere, suggesting local clustering (Vornoi maps, Fig. 6a–h). These observations highlight that NA varicosities in the cerebellum exist in local clusters. However, to understand whether the effect of training altered these local clusters, we quantified the variation of the Voronoi polygon sizes by calculating the coefficient of variation (COV) of polygon areas.

The COV of Voronoi polygon sizes revealed a 47 % variation for CON of both vermis and the hemisphere, demonstrating that even CON rats exhibit local clustering in the NA varicosity population, regardless of the cerebellar zone (Fig. 6j). Following voluntary exercise, the COV in the vermis was 36 % for the EX group, 37 % for the FR group, and greatly augmented to 75 % for the EEX group. The COV in the hemisphere was greatly reduced to 17 % for FR rats, indicating less clustering of varicosities for the FR group. To test whether there was an effect of training on the degree of clustering of NA varicosities residing in the vermis and the hemisphere, we performed an ANOVA on the ratio of the absolute deviations adjusted for the mean of each respective group. Since the Levene’s *F* test for COVs was not statistically significant, we conclude that, although there are clear local clusters of NA varicosities as observed from the Voronoi maps (Fig. 6a–h), the differences in COV are likely due to individual animal’s differences in local clustering, combined with the effect of training. To test this, we next conducted correlation analysis between individuals’ WRA and NA axon structure for each group.

### Individual running activity and NA plasticity

Food restriction-evoked running exceeded the extent of running by wheel access alone (Fig. 1b). However, closer examination of EEX group’s WRA indicated that excessive exercise was evoked by some, but not all animals of the EEX group. This individual difference in WRA within the group prompted us to ask whether individual differences in food restriction-evoked WRA might be associated with individual differences in NA axons’ structural plasticity. We first asked whether there existed an association between the amount that animals ran and inter-varicosity intervals along NA axons.

**Fig. 7:**
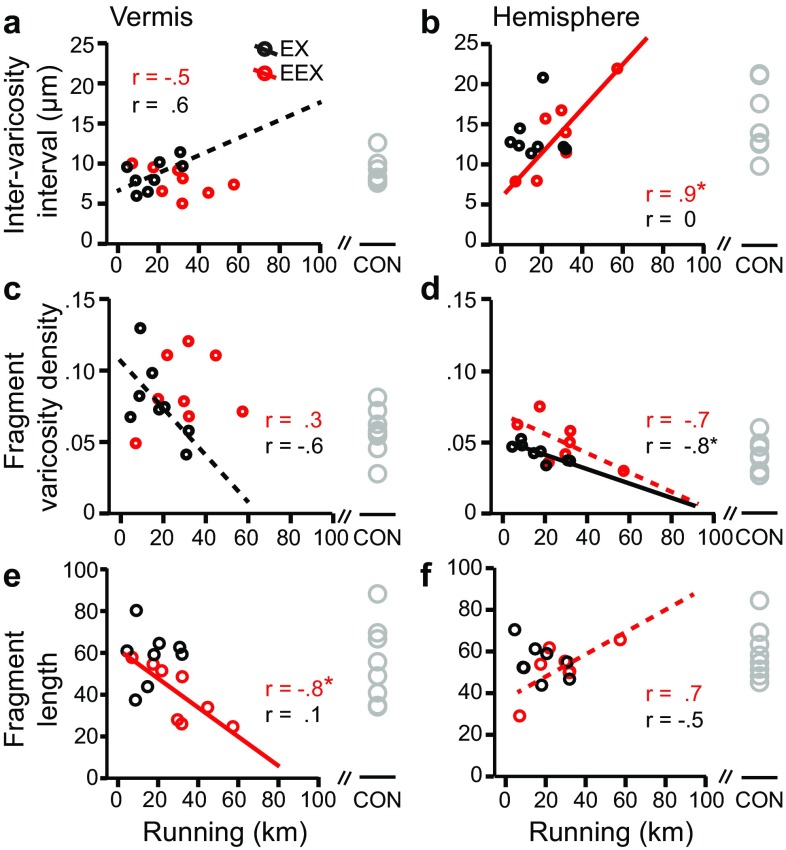
Correlations between WRA and NA varicosity distribution is dependent on anatomical region and running intensity. **a**, **b** Correlations between total WRA and the NA inter-varicosity intervals. **c**, **d** Correlations between total WRA and varicosity density along NA fibers or **e**, **f** NA fragment length. Correlation coefficients are designated by r values. Asterisks indicate statistically significant correlations (*P* < 0.05). Solid trend lines accompany strong and statistically significant correlations, while dotted trend lines indicate moderate correlations (r = ±0.6 or greater). *N* = 8 rats per group except *N* = 7 for the hemisphere of EEX

**Fig. 8:**
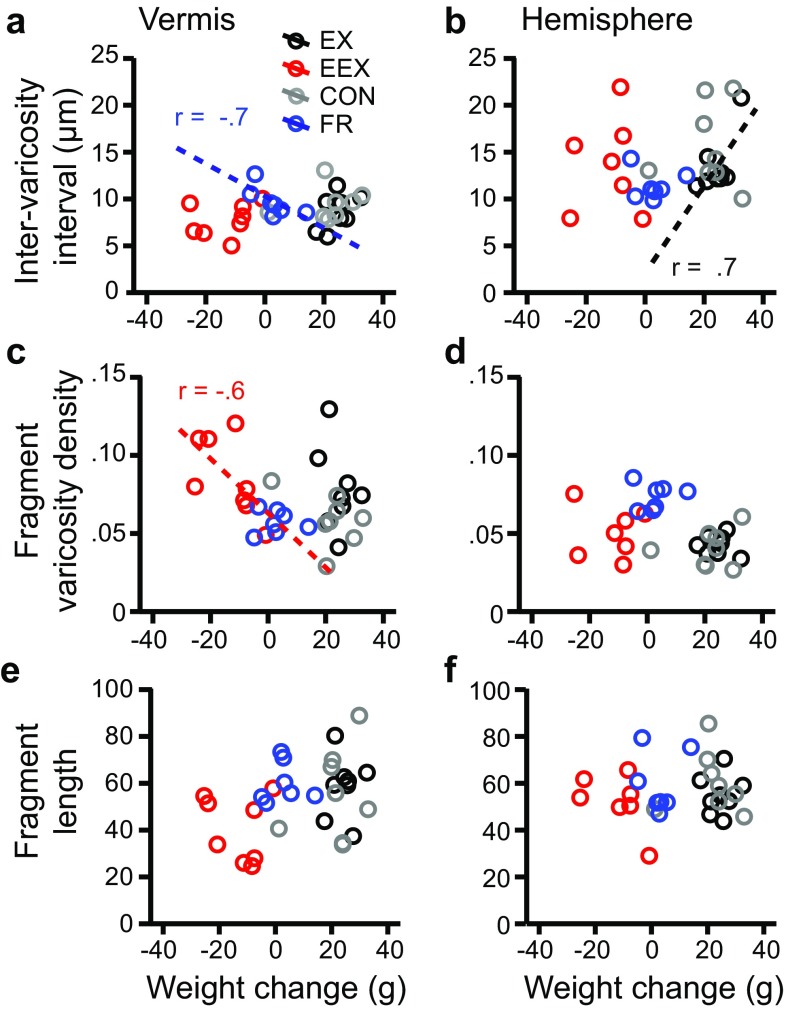
Correlations between weight change and NA plasticity. **a**, **b** Correlations between total weight change and the NA inter-varicosity intervals. **c**, **d** Correlations between total weight change and varicosity density along NA fibers. or **e**, **f** NA fragment length. Correlation coefficients are designated by *r* values. *Asterisks* indicate strong and statistically significant correlations (*P* < 0.05). Dotted trend lines indicate moderate correlations (r = ±0.6 or greater). *N* = 8 rats per group except *N* = 7 for the hemisphere of EEX and FR groups

In the vermis, EX rats showed a marginal correlation between total WRA and NA plasticity (Fig. 7a, c, r (8) = 0.6, *P* = 0.12 for inter-varicosity interval; r (8) = −0.6, *P* = 0.09 for fragment varicosity density). These results are consistent with one another, indicating that the more active EX individuals exhibited varicosities that were farther apart from one another along NA axons. However, in the vermis, no correlation between an individual rat’s total running activity and NA varicosity distribution along axons was detected for the EEX group (Fig. 7a). There also was no correlation between EEX animals’ total WRA and axon varicosity density in the vermis (Fig. 7c). The loss even of a weak correlation for the EEX group, when compared to the EX group, indicates that the extent of increase in axon fragment varicosity density among the EEX group (Fig. 7c) and decrease in the inter-varicosity intervals (Figs. 3a, 7a) were not due in any direct way to the extent of food restriction-evoked increase in running. There also was no direct relationship between NA rearrangements of inter-varicosity intervals with the extent to which the EEX rats lost body weight (Fig. 8a), while the varicosity density along NA fragments were weakly correlated with weight loss (Fig. 8c, r (8) = −0.6, *P* = 0.09). In contrast, the more that the EEX rats ran, the shorter were the NA axon fragments in the vermis, and this association was statistically significant (Fig. 7e; r (8) = −0.8, *P* < 0.05).

Comparisons of the correlations across the two cerebellar regions revealed an unexpected difference. The hemisphere, unlike the vermis, exhibited strong correlations between inter-varicosity intervals and the extent to which EEX animals ran. The correlation was positive (Fig. 7b, r (7) = 0.9, *P* < 0.05), meaning that the more that animals suppressed excessive WRA, thereby minimizing excessive weight loss (Fig. 1f, r (7) = −0.8, *P* < 0.05), the closer were the varicosities to one another along NA axons. This relationship was corroborated by the varicosity density along axon fragments, which showed a trend for a negative correlation (Fig. 7d; r (7) = −0.7, *P* = 0.1). The relationship between running and varicosity density along NA axon fragments was evident even without food restriction, since tissue from the EX group of animals exhibited an even stronger negative correlation between WRA and fragment varicosity density (Fig. 7d) (r (8) = −0.8, *P* < 0.05). Conversely, the weight change associated with food restriction-evoked hyperactivity did not contribute towards the structural changes of NA axons in the hemisphere, based on the lack of correlation between the two variables (Fig. 8b, d, f).

**Fig. 9:**
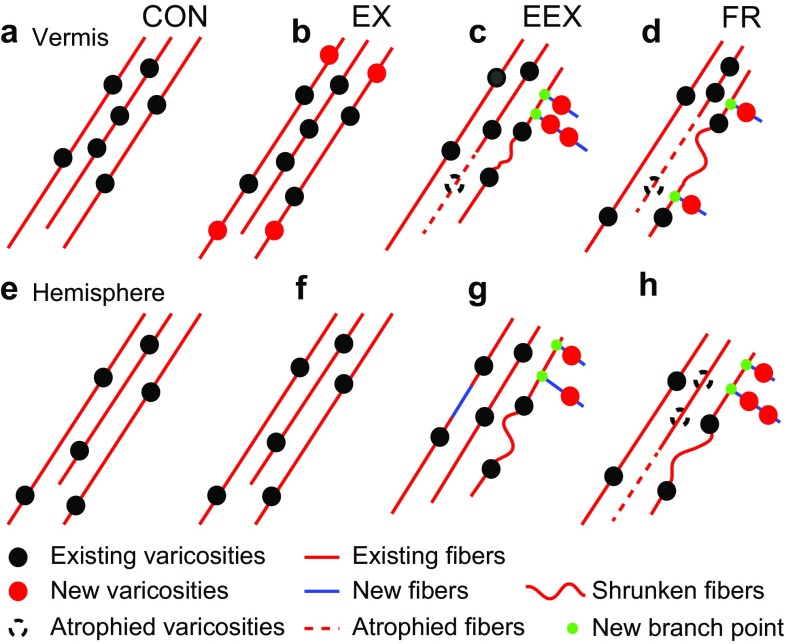
A neural model of exercise-evoked NA plasticity in the cerebellar vermis and hemisphere that is consistent with the collected data. **a**–**d** Vermis NA fibers and varicosities. **e**–**h** Hemisphere NA fibers and varicosities. **a**, **e** Intervals between varicosities for CONs are slightly shorter in cerebellar vermis, compared to cerebellar hemisphere (*P* < 0.01, Student’s *t* test), but both regions still exhibit similar levels of clustering (*P* > 0.05, Student’s *t* test). **b**, **f** Mild exercise-evoked addition of NA varicosities to existing fibers in the vermis of EX rats, but not in the hemisphere of EX rats. **c**, **g** Relative to CON rats, food restriction-evoked excessive exercise leads to shorter inter-varicosity intervals in both the vermis and the hemisphere of EEX rats. There is a higher frequency of shorter varicosity containing fragments especially in vermis of EEX rats, corroborating the increased clustering among the NA varicosity population. **c**, **g** and **d**, **h** Food restriction, with or without exercise, increases the proportion of short NA axon fragments in both the vermis and hemisphere. This change could be due to sprouting of new fibers (*blue*) associated with the emergence of new branch points (*green filled circles*) that are not captured in the sampled volume, atrophy of existing axons (*dotted red lines*) or shrunken fibers (*wriggly lines*). Red circles represent newly added varicosities. *Dotted circles* represent varicosities lost

There was a trend for a correlation between the hemisphere’s axon fragment lengths to running by the EEX group (Fig. 7f, r (7) = 0.7; *P* = 0.07): this relationship was reversed from that found for the vermis. In the hemisphere, the high runners of the EEX group responded with NA axon elongation. The elongated NA axons among the high runners of the EEX group most likely did not carry new varicosities, since the fragment varicosity density was lower (Fig. 7d) and the inter-varicosity interval was greater (Figs. 7b, 9). The lower runners among the EEX group may have responded with NA axon sprouting of varicosity-bearing NA axons or shrinkage of existing NA axons, as indicated by the emergence of average NA axonal fragments that were shorter than those of the EX group (Fig. 7f) but of higher varicosity density along NA fragments (Figs. 7d, 9).

These results reveal two points: (1) both mild and excessive exercise yield differences in NA fiber architecture that are detectable; (2) exercise alters NA axon structure in different ways across the vermis and the hemisphere and across animals in ways that correlate with their responsivity to the combination of food restriction and exercise.

## Discussion

The existence of NA axons in the cerebellum has been known for a long time (Hokfelt and Fuxe 1969), but our study is the first to indicate that just 8 days of voluntary WRA evokes structural plasticity of the NA system in the cerebellum. The nature of this plasticity differs between the vermis versus the hemisphere. Moreover, the NA fibers are already different across the two cerebellar regions, even in the cerebellum of sedentary animals. We discuss below about the functional implications of this regional difference.

### The vermis responds to mild and excessive exercise with structural changes reflecting increases of NA releasable sites

NA fibers in the molecular layer of the anterior vermis of both the EX and EEX group of rats show shortened distances between varicosities with immunoreactivity to DβH, the norepinephrine-synthesizing enzyme, compared to those of the CONs and FRs. This difference indicates a change evoked by running (Fig. 3b). The shortened distance indicates greater density of potential norepinephrine release and synthesis sites, thereby increasing the potential for the NA system to improve the signal-to-noise ratio of cerebellar afferents during states of arousal (McElligott et al. 1986). Functionally, the vermis plays a role in motor control, due to its direct connectivity to the motor cortex and inputs from the spinal cord (Sengul et al. 2014; Coffman et al. 2011; Provini et al. 1968; Strata et al. 2012; Voogd 2014), since spinocerebellar mossy fiber afferents terminate primarily in the anterior and posterior vermis region (Nieuwenhuys et al. 2008; Sengul et al. 2014). Our results are in agreement with studies that attribute “motor” (whole body posture and locomotion) function primarily to the vermis (Coffman et al. 2011; Voogd 2014) and reveal that the NA-mediated modulation of the spinocerebellum can be changed by the addition of exercise to daily activities. Based on our findings, one would predict that cerebellum-dependent motor tasks with dependence on NA afferents, such as a rat’s ability to learn to run on a rod runway (Watson and McElligott 1984) or the delay eyeblink conditioning (Paredes et al. 2009) will be enhanced by exercise. This rapid response (8 days of exercise) may be specific to adolescence. Further studies are required to determine whether NA fibers in the vermis respond to exercise in adulthood and pre-pubertally as well or as quickly.

For the EEX group, we detected an additional effect in the vermis: a negative correlation between NA fragment lengths and individual animals’ WRA, suggesting that animals that ran more exhibited shorter NA fragment lengths (Fig. 7e). This shortening of NA fragment lengths must have contributed to the leftward shift in the cumulative frequency of NA fragments in the EEX brains (Fig. 4e). The emergence of shorter NA fragments, especially among the most hyperactive individuals, may reflect the growth of new NA axon branches associated with increased exercise, the development of coordination and balance to sustain especially high levels of locomotive activity, and increased NA modulation of blood flow, since high level exercise is associated with angiogenesis (Black et al. 1990). We cannot rule out the alternative interpretation—namely that EEX caused shrinkage of NA axons (Fig. 9). Neither the FR nor the EEX group exhibited NA fragment lengths that related to their weight loss (Fig. 8e), indicating that the structural changes in the NA fibers is not due simply to starvation. Since the extent of food restriction-evoked WRA correlates with behavioral measurements of anxiety by the elevated plus maze (Wable et al. 2015), the apparent sprouting/atrophy could be an indirect effect arising from the food restriction-evoked rise of anxiety. The reason that the EX group did not exhibit this additional effect of shortened NA fragment lengths in the vermis may be that a longer duration of mild exercise is needed to evoke anatomically detectable changes, or that individual differences in voluntary WRA was too large for detecting the relatively smaller effect of mild exercise. We did observe a strong correlation between the distance run by the EX group of animals and varicosity density along NA fragments in the hemisphere (Fig. 7d), indicating that our approach was able to detect regional differences in NA structural plasticity.

NNVDs of the EX group are less than that of the CON’s in the vermis, while the EEX tissue did not exhibit a decrease of NNVD in the vermis. This lack of change in NNVD by the EEX tissue could be due to a combination of two counter-balancing changes: addition of new varicosities and sprouting, which would decrease NNVD, while atrophy of NA axons would increase NNVD, due to the loss of some neighboring varicosities (Fig. 9).

### Up-regulation of the NA varicosities in the hemisphere is associated with resilience to ABA, namely suppression of food restriction-evoked hyperactivity

The two most robust effects of EEX were detected in the hemisphere. They were (1) a decrease in the inter-varicosity interval along NA fibers (Fig. 3c) and (2) the strongly positive correlation between WRA and inter-varicosity intervals (Fig. 7b). These two measures depict a consistent relationship between NA axon structure and the food restriction-evoked hyperactivity and support the following interpretation—that addition of NA varicosities to NA fibers is related to the suppression of food restriction-evoked hyperactivity. Conversely, individuals that failed to add varicosities along NA axons were the ones that became excessively hyperactive following food restriction, thereby becoming voluntary food restrictors that choose to run on the wheel relatively more, even during the hour of food availability, thereby losing the greatest amount of body weight. Since we cannot know whether the individual differences in the inter-varicosity intervals existed prior to food restriction and wheel access or that they emerged as a consequence of treatment, our results from correlation analysis (Fig. 7b) also support the idea that individual differences in NA structure influenced the extent to which food-restricted individuals engaged in voluntarily exercise. Either way, the very strong correlation supports the idea that the shorter distances of inter-varicosities, which translates to greater potential NA release sites in the hemisphere, contributed towards suppression of excessive exercise.

While hyperactivity in the form of foraging is a natural response for animals facing starvation, suppression of hyperactivity is more adaptive when food restriction occurs within the confinement of a cage, in that suppression of hyperactivity minimizes weight loss and negative energy balance (Gutierrez 2013) and our Fig. 1f). The current data support the idea that animals with greater levels of NA varicosities in the hemisphere were the ones that could adapt more effectively by minimizing excessive running and weight loss. Since the hemisphere is interconnected relatively more with non-motor areas, including the prefrontal cortex (Strick et al. 2009), the work presented here points to a potentially novel role for the NA system in the cerebellar hemisphere—i.e., to improve an individual’s behavioral flexibility and coordination of motor control, such as wheel running, with other needs of the individual, such as conservation of energy, when placed under conditions of limited food supply and unsuccessful foraging. Recently, it was shown that the cerebellum modulates prefrontal cortical activity (Watson et al. 2014). Facilitated connectivity of the cerebellar hemisphere with cortical regions, especially the prefrontal cortex, via the NA system may be a circuit that helps individuals make better behavioral choices for survival, when faced with the decision to continue running on the wheel or eat.

We are not aware of any studies on the cerebellar NA system of patients with anorexia nervosa. Such a study would be worthwhile to address, given the observations of the cerebellum of EEX animals. The studies we have found relate only tangentially to this question. Plasma levels of norepinephrine among 7 patients with anorexia nervosa was lower (Obarzanek et al. 1994) and remained low for 2 individuals, together with lowered levels in the cerebrospinal fluid (CSF), even after weight restoration (Kaye et al. 1985). How much of the alteration in CSF level reflects changes in the brain is unclear, but given the contrasting responses across the two sub-divisions even of a single brain region, such as the cerebellum, it is possible that important regionally specific changes cannot be captured by CSF analysis. In support of the idea that central NA system is altered by the physiological condition of anorexia nervosa, major depression, a co-morbidity of anorexia nervosa that is linked, in part, to norepinephrine depletion centrally, usually emerges a year or longer after the onset of anorexia nervosa (Pirke 1996). This pattern fits with our observation in the hemisphere, where augmentation of the NA system is associated with resilience to ABA. An fMRI study indicates that processing of non-self body image activates the cerebellum, but that the extent of activation of the cerebellum is similar across patients with anorexia nervosa and healthy controls (Sachdev et al. 2008). This point is relevant for understanding the etiology of anorexia nervosa, since body dysmorphia is another core symptom of this disease, but not likely to be translatable from rodent models. The cerebellum exhibits reduced regional cerebral blood flow within brains of patients with anorexia nervosa, but only after weight restoration (Kojima et al. 2005). Authors do not provide an interpretation of this finding. Finally, focal atrophy in cerebellum was detected within brains of patients with anorexia nervosa (Boghi et al. 2011). For all of these studies, it is not possible to know whether the differences from healthy controls were causal to the condition of anorexia nervosa or reflect the aftermath of the altered nutritional and activity levels associated with anorexia nervosa. More work that compares NA modulation of cerebellum-dependent tasks of individuals with anorexia nervosa, recovered individuals, and healthy controls engaged in varying levels of exercise would be valuable for understanding the relationship between the cerebellar NA system and vulnerability to anorexia nervosa.

### Higher NA innervation is found in the cerebellar vermis

While comparable axon fragment lengths were encountered in the vermis and hemisphere (Fig. 5d), we found significantly shorter inter-varicosity and NNVDs values in the vermis of CON rats (Fig. 5a, b). The axon fragment varicosity density was also higher in the vermis (Fig. 5c). These results indicate greater potential sites for the release of norepinephrine in the vermis than in the hemisphere of sedentary rats. Greater NA innervation was found in the vermis of *Dbh*-*Cre_KH212* mice injected with anterograde adeno-associated viral (AAV) tracer in the LC (Allen Institute Connectivity Atlas, Online Resource 3), suggesting that the cerebellar vermis of rodents receive more NA innervation than in the hemisphere. A recent study injected a retrograde viral tracer in the LC of *Dbh*-*Cre* mice and found labeled Purkinje cells enriched in the ipsilateral medial zone (vermis) of the cerebellum (Schwarz et al. 2015). Taken together, these three lines of research demonstrate experimental evidence for a greater reciprocal connectivity between the LC and cerebellar vermis, compared to the hemisphere. The functional implication of a greater NA input to the vermis and less innervation to the hemisphere is an important topic to explore in future studies.

From our behavioral data, we also found shorter NNVDs, shorter inter-varicosity intervals and a greater density for varicosity density per axon fiber in the vermis post mild WRA, compared to the hemisphere. These results are similar to CON rats’ and recapitulate the finding of a higher NA innervation to the vermis already existing prior to mild exercise. In contrast, individuals that experienced food restriction-induced hyperactivity showed shorter NNVDs and a greater varicosity density along axons in the vermis, but no longer any regional difference in inter-varicosity intervals, when compared to the hemisphere. This equalization of potential sites for NA release across the two regions may be attributed to the effect of food restriction, which, alone, had opposite effects in the vermis versus the hemisphere (Figs. 4e, f, 9). In the vermis, the effect of food restriction may have counteracted the effect of exercise, while in the hemisphere the effect of food restriction was manifested without abatement of the exercise effect. Food restriction elicited opposite changes across the two regions: increase of inter-varicosity intervals along NA axons in the vermis and reduction of these measures in the hemisphere. In addition, food-restricted sedentary animals showed lower varicosity density along axon fibers in the vermis than in the hemisphere. These comparisons reveal that food restriction was influential in lowering the vermis-to-hemisphere difference in inter-varicosity distance along NA axons.

### Varicosities and norepinephrine release

Although we propose that varicosities are sites of norepinephrine release and local synthesis of norepinephrine, an alternative interpretation is possible. An intriguing point of norepinephrine released from sympathetic nerves is that a small portion escapes vesicular monoamine transport, and this rate of leakage exceeds exocytotic release at resting conditions (Eisenhofer et al. 2004). However, such non-vesicular release would not be synchronized with neuronal firing, while vesicular release would be strongly synchronized with neuronal firing and also with motor behavior involving cerebellar control. This is because vesicular release is linked to calcium influx via the voltage-gated calcium channels that open following neuronal firing in the LC, thereby generating phasic release of norepinephrine (Glaser et al. 2007), while leakage, by definition, would not be expected to be locked temporally to action.

Extensive serial reconstruction of NA varicosities in the cerebral cortex indicate that varicosities contain large numbers of vesicles, together with mitochondria, and that nearly all of them are associated with conventional synapses, while inter-varicosity segments contain only low densities of vesicles (Parnavelas et al. 1985; Papadopoulos et al. 1987a, b). Although the ultrastructure of NA varicosities in the cerebellum does not indicate such high occurrence of synaptic junctions (Abbott and Sotelo 2000; Sotelo 2011), these varicosities also are packed with vesicles and are supported by mitochondria, while the inter-varicosity portions exhibit only low densities of vesicles. These ultrastructural descriptions indicate that varicosity counts relate to the local availability of vesicularly releasable norepinephrine and energy supplied by mitochondria, even if they are not associated with postsynaptic junctions. Can vesicles within non-junctional varicosities actually release norepinephrine? Although this study did not address this question, much of the work on synaptogenesis indicates that varicosities attain release capability prior to the formation of stable axo-dendritic synaptic junctions. Such findings are based on direct visualization of vesicular exocytosis–endocytosis and vesicle aggregation within portions of glutamatergic axons lacking targets (Kraszewski et al. 1995; Krueger et al. 2003) and through detection of the quantal release of acetylcholine from axons devoid of target muscles (Hume et al. 1983; Sun and Poo 1987; Young and Poo 1983; Zakharenko et al. 1999). The idea of volume transmission, where norepinephrine is released from non-junctional varicosities, is not new (Descarries et al. 1975; Fuxe et al. 2010; DeFelipe and Jones 1988), as has been proposed for other monoamines as well (Parnavelas et al. 1985; Groves et al. 1994; Arbuthnott and Wickens 2007). Immuno-electron microscopic localizations of β- and α-adrenergic receptors reveal that there are a large number of non-junctional ARs (α and β) (Aoki et al. 1998; Aoki 1992, 1997), suggesting that NA axon terminals can generate specific physiological responses that depend on where the ARs are present, regardless of whether the varicosities are junctional or not.

### The potential impact of food restriction upon the NA structural plasticity and motor learning

Bickford and colleagues have shown that food restriction by 40 % over a period of 10 months or longer (as opposed to 75 % reduction over a 4-day period in our study) improves cerebellum-dependent motor skills of aged rats, without changes in the number or affinity of β2-adrenergic receptors (Gould et al. 1995). We observed NA varicosities become closer to one another following 4 days of food restriction alone and when combined with wheel access, specifically in the hemisphere (Fig. 4d). Whether or not the food restriction-evoked increase in the density of NA varicosities along NA axons of adolescent female rats is associated with improvements in the rate of acquisition or consolidation of cerebellum-dependent associative learning is another question never before posed but would be interesting to pursue.

### Stress

We have previously shown that the extent of WRA correlates with anxiety-like measurements in the elevated plus maze (Wable et al. 2015). Moreover, animals with food restriction-evoked hyperactivity (i.e., activity-based anorexia) have elevated activity of the hypothalamic–pituitary–adrenal axis and elevated levels of corticosterone (Burden et al. 1993). Based on this relationship, EEX is likely to be a manifestation of stress-evoked anxiety that leads to self-starvation (Gutierrez 2013), while suppression of excessive WRA is life-saving.

Several studies point to voluntary exercise exerting stress resilience (Cotman et al. 2007; Duman 2005; Greenwood and Fleshner 2008; Dishman 1997; Dishman et al. 2000). Voluntary exercise offers anxiolytic potential through a mechanism that enhances the expression of galanin, a peptide co-expressed with norepinephrine in the majority of LC cells and which suppresses NA output (Sciolino et al. 2012; Sciolino and Holmes 2012). These experiments show that three to 4 weeks of running reduces norepinephrine activity via changes in galanin mRNA levels. Intriguingly, our work shows evidence for NA plasticity in as few as 8 days and in the opposite direction, through increases in NA varicosities. This quick response of the NA system is not surprising since the LC, which is the source of norepinephrine to the cerebellum, is sensitive to stress response (Valentino and Van Bockstaele 2008). The agent that mediates activation of the LC during stress is the corticotropin-releasing factor (CRF), which elicits a stronger signaling cascade in females than in males (Valentino et al. 2013). Our study used female adolescent rats. Whether the same response would be observed for males or for younger or older females has yet to be determined. Nor do we know whether a longer duration of voluntary exercise will eventually reduce norepinephrine activity, as is reported by Sciolino and colleagues. Finally, although this study analyzed the NA axons in the cerebellum, it is expected that the EX, FR and EEX effects may be global, arising from changes at the transcription level of LC neurons. We are testing this idea by determining whether other NA target areas of the brain also exhibit changes in NA varicosity distributions in ways that relate to individual differences in the food restriction-evoked hyperactivity.

Although it is widely recognized that voluntary exercise promotes BDNF expression and secretion by excitatory neurons of the hippocampus (Rothman and Mattson 2013; Cotman and Berchtold 2002; Neeper et al. 1996) a point relevant to this study is that this up-regulation of BDNF may require NA activation of β-adrenergic receptors [for review, (Ma 2008)]. A major source of BDNF in the cerebellum is also the excitatory afferents, namely the mossy fibers (Chen et al 2016). Although not yet established, it is likely that BDNF expression and secretion from mossy fibers would also be increased by exercise, since mossy fibers provide tactile input to the cerebellum activating restricted granule cells following mechanical stimulation of receptive fields on the body (Shambes et al. 1978). There is a circuit to support the recruitment of LC axons during exercise, as there is reciprocal connectivity between the cerebellum and LC (Schwarz et al. 2015; Hokfelt and Fuxe 1969). In the cerebellum, BDNF promotes the formation of GABAergic synapses surrounding glutamatergic axon terminals (Chen et al. 2016). Taken together, these studies call for new investigations on the important role of physical activity in mediating the NA- and BDNF-dependent synaptic plasticity in the cerebellum. Moreover, although exercise has been shown to promote synaptic plasticity and neurogenesis in the hippocampus, this beneficial effect is not seen or is delayed among rodents housed singly (Leasure and Decker 2009; Stranahan et al. 2006). It is possible that social isolation reduces the NA axons’ responses to exercise which, in turn, limits the promotive effects of BDNF. In the cerebellum, we observed EX to have stronger influence upon NA axons in the vermis than in the hemisphere, while EEX had strong influence upon NA axons of both the hemisphere and the vermis. Ultimately, these differential responses of the NA system across the two regions of the cerebellum to exercise may translate to differences in the up-regulation of BDNF by exercise across the two regions, leading to greater synaptic plasticity of the vermis under conditions of mild exercise and plasticity of the hemisphere only under conditions of extreme exercise. These are testable working hypotheses.

## Electronic supplementary material

Below is the link to the electronic supplementary material.

Supplementary material 1 (DOCX 1553 kb)

Supplementary material 2 (MOV 8917 kb)
