# Supplementary material for: Cerebellar sub-divisions differ in exercise-induced plasticity of noradrenergic axons and in their association with resilience to activity-based anorexia
Source: Brain Struct Funct. 2016 Apr 7;222(1):317–39. doi: 10.1007/s00429-016-1220-2 (PMC5215061; doi:10.1007/s00429-016-1220-2)

Cerebellar Sub-divisions Differ in Exercise-Induced Plasticity of Noradrenergic Axons and in their Association with Resilience to Activity-Based Anorexia

**Hermina Nedelescu^1,2,3^* Tara G. Chowdhury^2^, Gauri S. Wable^2^, Gordon Arbuthnott^3^ and Chiye Aoki^2^***

^1^Department of Systems Neurophysiology, Tokyo Medical and Dental University Graduate School, 1-5-45, Yushima, Bunkyo-ku, Tokyo, Japan, 113-8519

^2^Center for Neural Science, New York University, New York, New York, 10003

^3^Brain Mechanisms for Behaviour Unit, Okinawa Institute of Science and Technology Graduate University, Okinawa, Japan, 904-0495

*****To Whom Correspondence May be Addressed:

Hermina Nedelescu

Department of Systems Neurophysiology

Tokyo Medical and Dental University

Tokyo, Japan

Email: [hn435@nyu.edu](mailto:hermina.nedelescu@oist.jp); hermina.nedelescu@gmail.com

Chiye Aoki

Center for Neural Science, New York University

New York, NY 10003, USA

Email: [ca3@nyu.edu](mailto:ca3@nyu.edu)

**Online Resource 1:**

Wheel Running Activity. Total running activity broken down day-by-day starting with Day 2. Note the increase in running activity for both EX and EEX animals after Day 4, with significantly more increase in running behavior for the EEX group (asterisks indicate *P* < 0.01, N = 8 animals per group). Error Bars: ± 1 SEM.


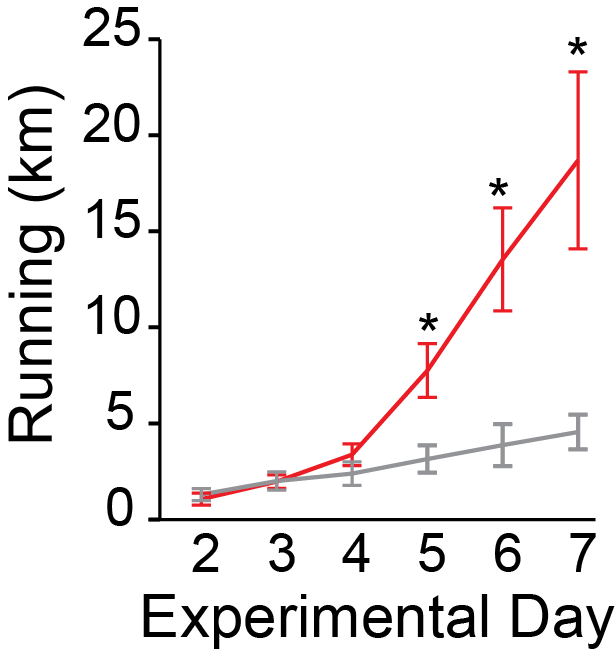


**Online Resource 2:** (Still image for supplementary video)

Three-dimensionally reconstructed NA varicosity axons. Video first demonstrates a raw image of DβH-immunoreactive NA axons in the cerebellum. Subsequently, the reconstructed axons in the molecular layer (red), Purkinje cell layer (purple) and granule layer (black) appear with the location of their respective black, orange and green markers, reflecting NA varicosities. Note that marker size does not reflect actual varicosity size. Even though all axons were reconstructed, not all axons are visible at this level of depth in the 2D raw image. The Purkinje cell layer is delineated by the flat contours around the Purkinje cell bodies, in gray. Lastly, the video rotates to show the 10μm thickness of the reconstructed NA axons in 3D. Image volume: 224μm x 224μm x 10μm.

**
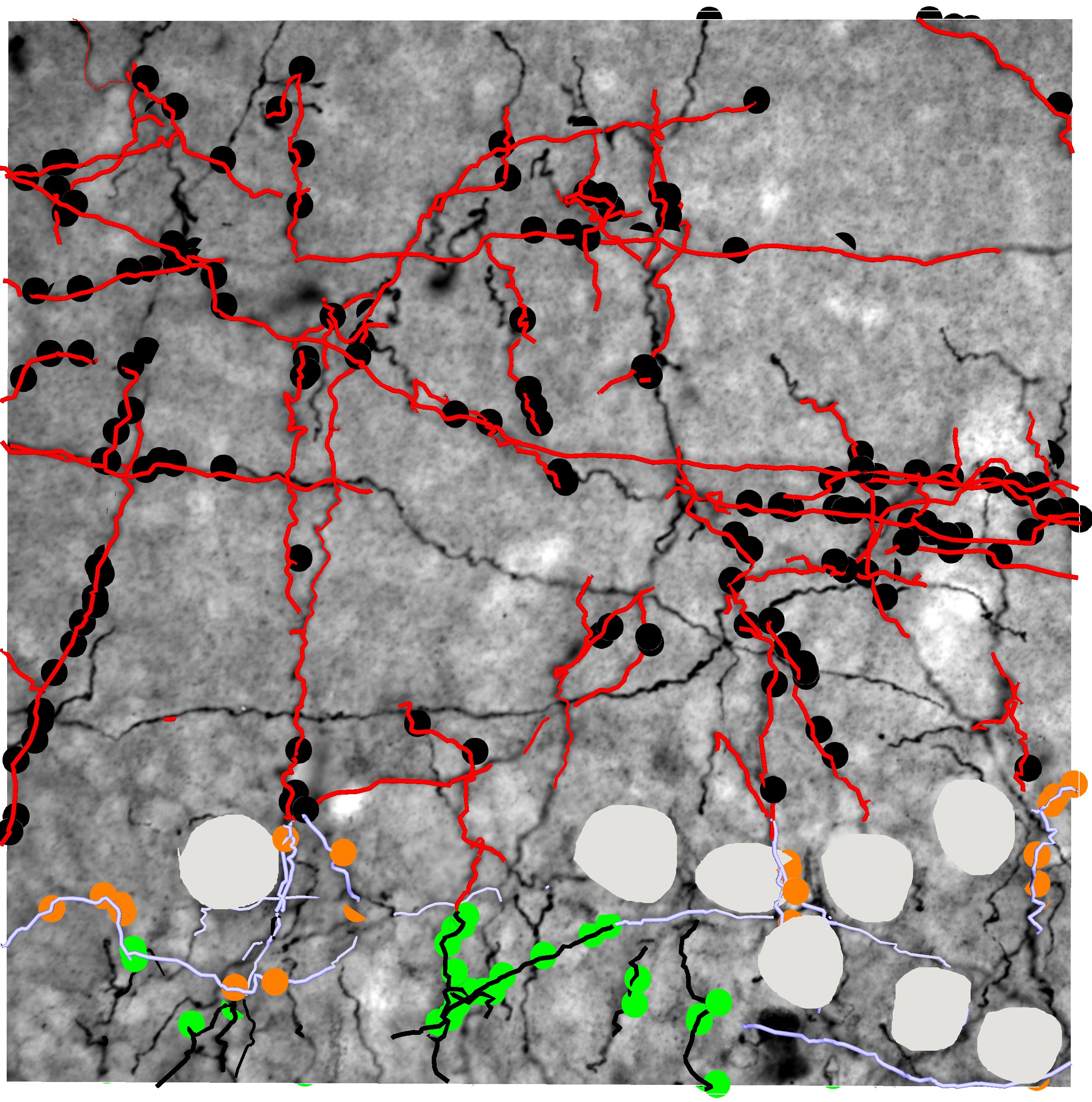
**

**Online Resource 3:**

Maximum intensity projections were composed from eight coronal images, comprising the anterior vermis. These images were downloaded from the Allen Institute Mouse Connectivity Atlas (http://connectivity.brain-map.org), depicting NA fibers (green) in the cerebellar cortex of a *Dbh-Cre* mouse injected in the LC with an anterograde tracer. Yellow boxes depict the hemispheres, while the purple box depicts the vermis with a higher density of NA input.


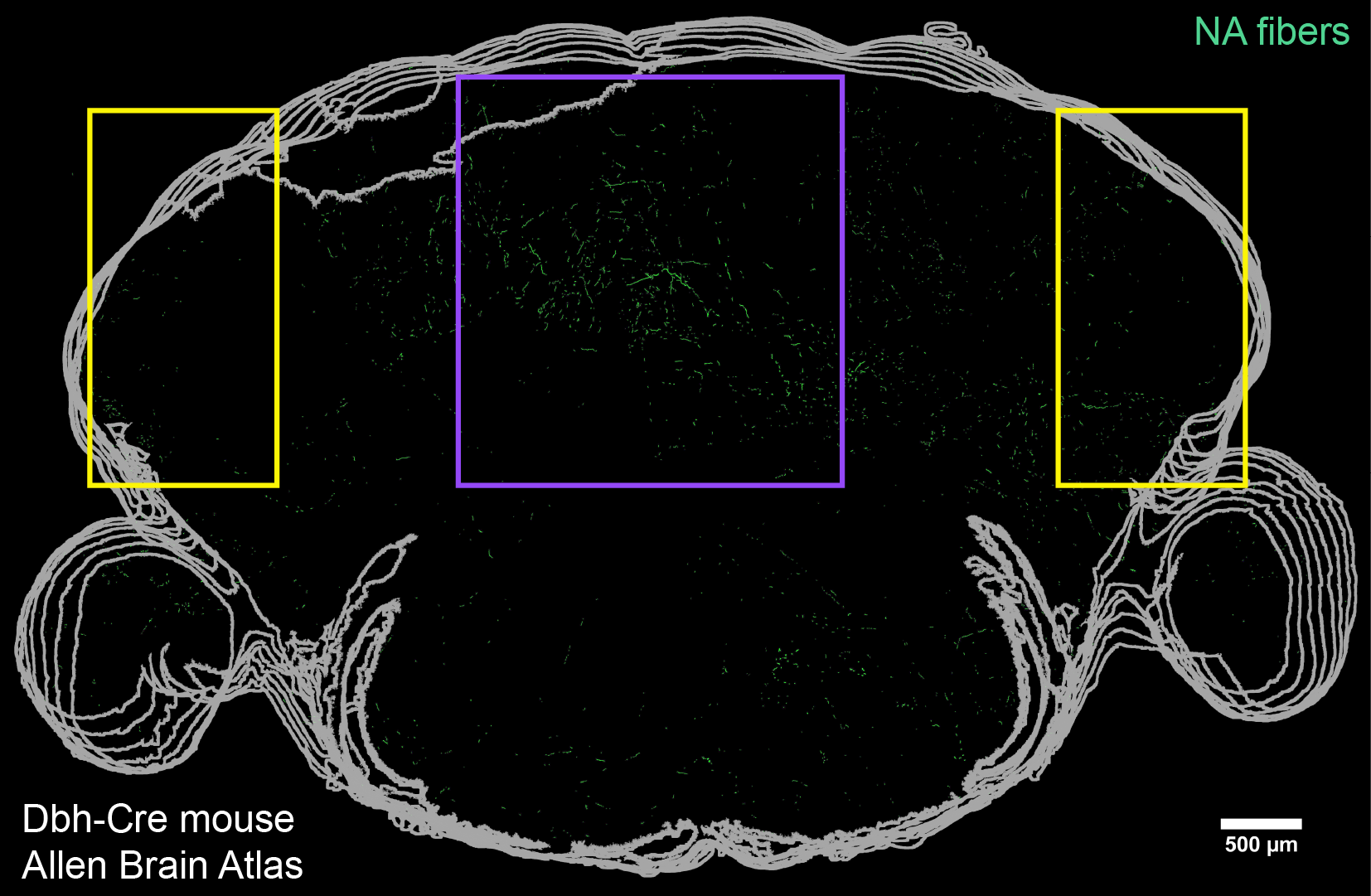

Supplement: Supplementary file 1 — Supplementary material 1 (DOCX 1553 kb) [file 429_2016_1220_MOESM1_ESM.docx]
